# EEG generation mechanism of lower limb active movement intention and its virtual reality induction enhancement: a preliminary study

**DOI:** 10.3389/fnins.2023.1305850

**Published:** 2024-01-30

**Authors:** Runlin Dong, Xiaodong Zhang, Hanzhe Li, Gilbert Masengo, Aibin Zhu, Xiaojun Shi, Chen He

**Affiliations:** ^1^School of Mechanical Engineering, Xi’an Jiaotong University, Xi’an, Shaanxi, China; ^2^Shaanxi Key Laboratory of Intelligent Robots, Xi’an Jiaotong University, Xi’an, Shaanxi, China; ^3^General Department, AVIC Creative Robotics Co., Ltd., Xi’an, Shaanxi, China

**Keywords:** movement intention, electroencephalogram, virtual reality induction, movement-related cortical potential, event-related desynchronization, brain-computer interface

## Abstract

**Introduction:**

Active rehabilitation requires active neurological participation when users use rehabilitation equipment. A brain-computer interface (BCI) is a direct communication channel for detecting changes in the nervous system. Individuals with dyskinesia have unclear intentions to initiate movement due to physical or psychological factors, which is not conducive to detection. Virtual reality (VR) technology can be a potential tool to enhance the movement intention from pre-movement neural signals in clinical exercise therapy. However, its effect on electroencephalogram (EEG) signals is not yet known. Therefore, the objective of this paper is to construct a model of the EEG signal generation mechanism of lower limb active movement intention and then investigate whether VR induction could improve movement intention detection based on EEG.

**Methods:**

Firstly, a neural dynamic model of lower limb active movement intention generation was established from the perspective of signal transmission and information processing. Secondly, the movement-related EEG signal was calculated based on the model, and the effect of VR induction was simulated. Movement-related cortical potential (MRCP) and event-related desynchronization (ERD) features were extracted to analyze the enhancement of movement intention. Finally, we recorded EEG signals of 12 subjects in normal and VR environments to verify the effectiveness and feasibility of the above model and VR induction enhancement of lower limb active movement intention for individuals with dyskinesia.

**Results:**

Simulation and experimental results show that VR induction can effectively enhance the EEG features of subjects and improve the detectability of movement intention.

**Discussion:**

The proposed model can simulate the EEG signal of lower limb active movement intention, and VR induction can enhance the early and accurate detectability of lower limb active movement intention. It lays the foundation for further robot control based on the actual needs of users.

## 1 Introduction

With the aging population and the frequency of accidents, the number of individuals with lower limb dyskinesia is gradually increasing. Exercise therapy after surgery has been indicated as an effective way to help patients recover (McDonald et al., 2022). Human lower limbs support the weight of the body, which creates movement challenges for lower limb dyskinesia individuals, so they inevitably need external assistance. Exoskeleton robots can not only help the body stand upright but can also assist the user in walking. It has been extensively researched, designed, and implemented (Kalita et al., 2020; Xiang et al., 2020).

Exoskeleton robots are required to be able to personalize and intelligently assist the user. An important principle within the use of exoskeleton robots is that the robots assist lower limb dyskinesia people to actively undertake prescribed movements rather than their limbs moving passively. It is critical that robots perceive the user’s movement intention (Qiu et al., 2021). Human active movement intention is the result of cognitive processes in the brain. The process of human brain cognition and its body expression can be described in two parts: “Uplink Pathway: Electroencephalogram (EEG) signal generation” and “Downlink Pathway: Electromyography (EMG) and other biological signal generation” (Zhang et al., 2021). EEG contains brain real-time information, which could be used to understand the current motor-related brain activity and further predict the next motor task. Furthermore, a brain–computer interface (BCI) could help researchers to investigate users’ movement-related neurophysiological changes in a non-invasive way (Abiri et al., 2019). This technology would pave the way for intelligent assistance of exoskeleton robots.

Some scholars have studied the neurophysiological changes related to movement intention through BCI. Detection or prediction of movement intention via EEG signals is the ultimate goal. Sburlea et al. (2017) investigated the detection of gait intention; the results showed that the detector combines movement-related cortical potential (MRCP) amplitude and phase features of EEG signals and has 62.5% accuracy in healthy subjects and 59% in stroke patients. Lopez-Larraz et al. (2016) identified movement intention with the cue-guided paradigm; the accuracy of movement intention detection was 84.44% in healthy subjects and 77.61% in incomplete spinal cord injury patients. Jeong et al. (2017) validated a single-trial readiness potential performance in the lower limb exoskeleton environment and the average classification accuracy was 80.7% in healthy subjects. Hasan et al. (2020) employed a discrete wavelet transform-based method to detect the movement intention, the accuracy of detecting “rest vs. start” was 76.41% and “walk vs. stop” was 74%, with healthy subjects outperforming amputee subjects. In addition, there are some studies on filtering algorithms (Jeong et al., 2020; Mascolini et al., 2022), feature extraction methods (Wang et al., 2020; Jia et al., 2022), and detection methods (Chaisaen et al., 2020) to improve the performance of movement intention detection or prediction. The accuracy of lower limb movement intention detection in people with dyskinesia is lower than in healthy individuals with BCI intention detection. How to enhance the detectability of lower limb movement intention in people with dyskinesia based on BCI and enable them to accurately control exoskeleton robots is an urgent problem.

To solve this problem, the process by which the brain generates movement intentions and related EEG signals should be analyzed. The brain can filter and select the information, and only selectively filtered information can be perceived (Leone et al., 2017). The brain’s generation of specific intentions is affected by facilitating and preventive factors, which is a process of competition. Individuals with lower limb dyskinesia experience physical discomfort during exercise, such as pain or fatigue, which generates a plethora of preventive factors that affect movement intentions. Although EEG signals have many advantages, they are inherently weak, non-stationary, and susceptible to interference. The brain of individuals with lower limb dyskinesia contains ambiguous and complex information, with a low signal-to-noise ratio and multiple confounding factors, which are not conducive to the analysis of movement-related neural signals (Jochumsen et al., 2015; Sburlea et al., 2016). Therefore, their movement intention detection accuracy is lower than that of healthy people. However, the current BCIs cannot address the impact of multiple factors on people with lower limb dyskinesia.

Cognitive neuroscience research suggests that human movement intention and preparation are greatly affected by their mental state of exercise (Van Overwalle et al., 2020). Some scholars have proposed to introduce virtual reality (VR) technology in rehabilitation training to build a three-dimensional audio-visual integrated virtual environment with multiple perceptions so that users can complete the two-way interaction between virtual and reality in a simulated environment. Immersive scenarios could help to filter out some of the external distractions and maximize the user’s ability to focus on the movement task, promoting motor neurological rehabilitation. Berton et al. (2020) statistically analyzed the clinical data on the impact of VR technology on orthopedic patients from 2015 to 2020, the results showed that VR technology had a positive effect on the rehabilitation of patients. The case study by Chillura et al. (2020) showed that the combination of traditional rehabilitation under VR and robot-assisted rehabilitation could enhance functional recovery; the improvement effect of patients after combined treatment was significantly greater than that after conventional rehabilitation alone. Maggio et al. (2021) evaluated the usefulness of robot-aided gait training (RAGT) equipped with virtual reality augmented visuomotor feedback through EEG changes and confirmed that RAGT and VR can achieve better patient-tailored improvement in functional gait. These studies showed that VR technology plays a positive role in the rehabilitation of people with lower limb dyskinesia, but this is only a qualitative description, which does not indicate the impact of VR on brain or body changes in movement. Whether VR technology enhances movement intention or improves the detectability of movement intention is still unclear, and understanding the effect of VR on movement-related EEG potential change is important for movement intention detection based on BCI.

To study the mechanism of VR induction, it is necessary to analyze the brain information processing, establish a model for the generation of EEG signals, and then analyze the effect of different situations on its signal features. Some mathematical models of EEG generation were established and EEG signals have been simulated. Wendling et al. (2002) focused on the high-frequency EEG activity and modeled EEG signals in epileptic patients. Zavaglia et al. (2008) built an EEG generation model by combining three neural mass models and simulated EEG power spectral density in some regions of interest during simple tasks. Chehelcheraghi et al. (2017) added external modulatory input and dynamic self-feedback to the Wendling neural mass model and simulated EEG signals in α, β, and γ bands. These models simulated macroscopic EEG signals by considering the brain regions of the sensing zone as a whole. However, brain information processing involves different regions and has a hierarchical relationship. Moreover, elements related to movement intention generation need to be added to the model to produce the corresponding EEG signal features. In this regard, the van der Pol oscillator is often used as a model for simulating EEG features. Baghdadi et al. (2018) modeled the ERD/ERS features of EEG signals using van der Pol oscillator simulations. Ghorbanian et al. (2015) simulated eyes-closed and eyes-open EEG based on the van der Pol oscillator, and the model showed that very good agreement exists between the model output and the EEG in terms of the power spectrum. Szuflitowska and Orlowski (2021) applied the Van der Pol model oscillator to study brain activity during temporal left lobe seizures. Therefore, to establish the EEG generation model of movement intention, it is necessary to combine the above different types of models and add more details. After modeling, the effect of the VR system on the EEG signal features can be analyzed by changing the parameters of the mathematical model.

In this study, we investigated the EEG generation of lower limb movement intention and its action expression mechanism and categorized brain information processing into primary and advanced processing. Then, an EEG signal generation model of lower limb active movement intention that fused the van der Pol oscillator and the neural mass model was established based on brain information processing laws. In addition, the model was simulated to investigate whether it is possible to enhance the movement intention significantly from EEG signals during leg lifts with VR induction. Finally, a comparative experiment was conducted on 12 healthy subjects to analyze EEG signal features and verify the correctness of the model. The rest of the paper is constructed in the following way. Section 2 describes the model and methods of the study. Section 3 describes the experiment. Section 4 contains the results of the model simulation and experiment. Section 5 is the discussion of the study. Section 6 is the limitations. Section 7 provides a conclusion to the study.

## 2 Methodology

### 2.1 Generation of lower limb movement intention and its virtual reality induction enhancement mechanism

**FIGURE 1:**
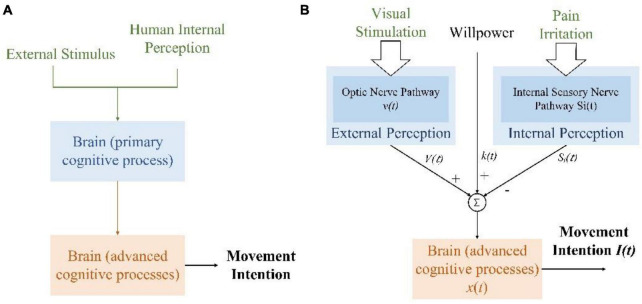
Generation of lower limb movement intention. **(A)** The generation of movement intention with secondary brain processing. **(B)** Various factors compete to produce movement intention for individuals with lower limb dyskinesia.

The brain generates intention after cognition and decision-making. The process of movement intention generation is shown in Figure 1A. Human receptors continuously receive information from the body’s internal or external environment, which is converted into electrical signals and transmitted to corresponding brain regions through specific nerve conduction pathways for primary processing. Then, the brain reprocesses and fuses the results with different primary processing regions, which can be regarded as an advanced processing process. Finally, the movement intention is generated and expressed in the EEG signals of the brain motor area.

For individuals with lower limb dyskinesia, pain during movement is the main factor that affects their movement intention. This is a prevention factor. However, psychological experimental research shows that visual information accounts for the largest proportion of the information received by the brain, which is 83% (An et al., 2017). In a VR induction system, virtual scenarios could provide an immersive and directional environment. It helps the users to perceive and focus on specific targets, generate selective attention, and enhance attention retention. Special scenarios could improve the competitiveness of the promoting factors for intention generation in the information-selective processing of the brain. In addition, individual willpower also affects the continuity of movement intention. The process of generating lower limb movement intention with the VR induction system is shown in Figure 1B. Pain irritation and individual willpower usually cannot be changed, while VR scenarios could provide positive visual information for generating movement intention and maximizing its benefits. Furthermore, attention enhancement and retention could enhance the efficiency of useful information, that is, enhancing motor control circuitry of the human brain, making movement-related neural associations easy to detect. Thus, the VR induction system increases the influence of specific signals in the brain’s advanced processing process by altering the input signals.

### 2.2 Mathematical models

Brain neurons encode movement intention. The generation of neural oscillations is considered a marker of brain activity, it can be analyzed by mathematical models of brain dynamics. Two types of brain dynamics models are commonly used to describe the generation of EEG signals. The first type is the micro-level model, which describes the activity of a single neuron in detail; explicitly combines the properties of ion channels, axons, and dendrites; and explores the chemical properties of action potentials with the changes in intracellular ion concentrations (Abbott, 1999; Baladron et al., 2012). The model is computationally complex and ignores the interactions between cells, which cannot fully reveal the response characteristics of whole brain EEG signals (Bossy et al., 2015). The second type is the neural mass model (NMM), which is proposed due to the discovery that neurons with the same function can cluster and have similar dynamic properties (Wilson and Cowan, 1973). It assumes that neurons in the same population share similar inputs and synchronize their activity, reflecting the overall discharge behavior by describing the average characteristics of the neural population (such as average discharge rate, average membrane potential, etc.). The model uses multiple state variables to describe the dynamics of the entire neural population and its synapses, which has the advantages of low computational complexity, simple parameters, and clear physiological significance.

According to physiological research and the foregoing analysis, there are two stages between receiving various information from human receptors and generating movement intention. The goal of the primary processing model is to synchronize the generated signals with the external stimulus signals. The advanced processing model couples the multiple types of signals obtained from the primary processing to output EEG signals indicating movement intention. Cascading these two models is the model proposed in this paper. In comparison with the universal model that simulates EEG at different frequencies, the proposed model could simulate not only spontaneous EEG signals but also the situation with external stimuli. Compared with the model that mimics the shape of EEG features, the proposed model has a clear physical significance. Overall, the proposed model is a model for movement intention generation that is more consistent with the physiological knowledge of brain processing and has good interpretability. The brain activity of movement intention generation could be expressed by the following two models.

#### 2.2.1 Brain primary processing model

With the development of physiology and anatomy, researchers have segmented the brain according to its cell composition, arrangement, structure, and other characteristics. Each region plays a different role in processing information in the brain. Rhythmic neural electrical activity is the basis of brain cognitive function. This spontaneous neural electrical activity can be regarded as a dynamic self-excited oscillation process. When stimulated by the external environment, the endogenous neural activity in the brain is regulated by the external stimulus, the neural oscillation will be synchronized with the external rhythm (Thut et al., 2011). Thus, in primary brain processing, the neurons could be represented as oscillators, and synchronization is the oscillatory output.

A small oscillating neuronal assembly could be described by a van der Pol oscillator, as shown in Equation 1. *Y* is the output, λ is the bifurcation parameter, and $\dot{Y}$ determines the state variable of the oscillator. When λ ≤ 0, there is no oscillation, when λ > 1, it enters a specific limit cycle with periodicity, and when 0 < λ < 1, the oscillator oscillates at a frequency *p* with an amplitude of $2\,\sqrt{\lambda }$.


(1) $$\ddot{Y}-\left(\lambda -Y^{2}\right)\,\dot{Y}+p^{2}\,Y=0$$

When an external input (including the external input from the VR induction system) has the same frequency as the oscillator, the neural population could be affected by the oscillation. When the oscillator receives an input of the same frequency, the oscillator would not be affected when the input is the same as the oscillator phase, and the oscillator could gradually converge to the input through periodic stimulation when the phase is different. As the period increases, the average phase of the neuron oscillator gradually converges with the input, which is synchronization. The completion of synchronization means that information has been transmitted to corresponding brain regions through specific neural pathways.

#### 2.2.2 Brain advanced processing model

The advanced processing of the brain is coordinated through the firing activity of a large number of widely interconnected neurons. It can be described in NMM. Based on the generation mechanism of nerve impulses, the classical NMM describes the interaction of different synaptic dynamics among pyramidal neurons, excitatory interneurons, and inhibitory interneurons (Wilson and Cowan, 1972; Jansen et al., 1993). It was initially used to study the mechanism of generation of alpha rhythms (Lopes da Silva et al., 1976) and the generation of simulated visual evoked potentials (Jansen and Rit, 1995). Physiological anatomy research suggests that the inhibitory synapses of pyramidal neurons in the hippocampus of the brain can be divided into slow inhibitory synaptic responses and fast inhibitory synaptic responses (Miles et al., 1996). Therefore, researchers divided inhibitory interneuron populations into slow inhibitory interneuron populations and fast inhibitory interneuron populations, and successfully simulated EEG signals that input targets were pyramidal cells (Wendling et al., 2002). However, input from neuronal populations can reach every interneuron. The results of a parameters sensitivity analysis showed that the model dynamics changes of excitatory interneurons and slow inhibitory interneurons were not obvious; thus, only the inputs to pyramidal neurons and fast inhibitory interneurons need to be considered. In addition, fast inhibitory interneurons exhibit self-inhibition (Ursino et al., 2010). Simulating brain parameters under different conditions (Vindiola et al., 2014; Mangia et al., 2017; Ferrat et al., 2018) and constructing a coupled brain network structure can clarify the changes in brain activities (Garnier et al., 2015; Chehelcheraghi et al., 2016).

**FIGURE 2:**
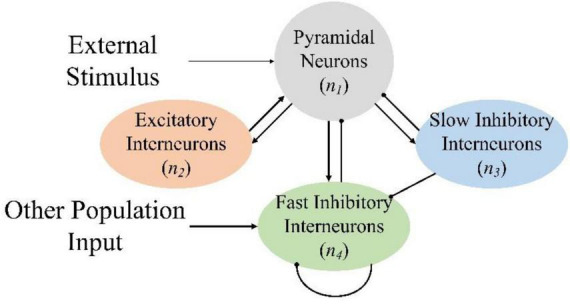
Neural mass model topology. The arrow line represents the excitatory transmission process, and the round-headed line represents the inhibitory process.

Based on previous research, the improved NMM topology is shown in Figure 2, where pyramidal neurons receive excitatory inputs from the excitatory interneurons and inhibitory inputs from the fast inhibitory interneurons and slow inhibitory interneurons. Pyramidal neurons transmit excitatory inputs to excitatory interneurons, fast inhibitory interneurons, and slow inhibitory interneurons. Slow inhibitory interneurons transmit inhibitory input to fast inhibitory interneurons and the fast inhibitory interneurons inhibit themselves. In the model, *n* is used to represent the neuron, and subscripts 1, 2, 3, and 4 of parameters represent pyramidal neurons, excitatory interneurons, slow inhibitory interneurons, and fast inhibitory interneurons, respectively. Attention enhancement and retention produced by the VR induction system affect the connectivity parameters of the various neuronal clusters, thereby enabling the brain to generate specific EEG signals.

#### 2.2.3 Lower limb movement intention generation model

**FIGURE 3:**
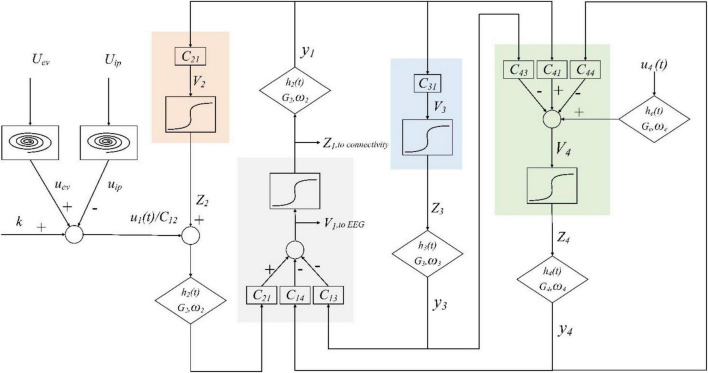
Layout of the lower limb movement intention generation model.

The primary processing and advanced processing of the brain are connected in series. According to the above method, the movement intention is generated when the promote factors are greater than the prevent factors; the motor areas of the cerebral cortex also display characteristic EEG signals. The model is shown in Figure 3.

In brain advanced processing, the primary processing results are collectively used as the input to the pyramidal neurons:


(2) $$u_{1}\,\left(t\right)=u_{e\,v}\,\left(t\right)-u_{i\,p}\,\left(t\right)$$

Where *u*_*ev*_(*t*) represents the result of the primary processing of positive external visual information *U*_*ev*_(*t*) and *u*_*ip*_(*t*) represents the result of the primary processing of negative internal pain irritation *U*_*ip*_(*t*). The primary processing is simulated by the van der Pol oscillator.

Fast inhibitory interneurons could also receive input from other populations, which is recorded as *u*_4_(*t*). Here, it is assumed that they are connected through excitatory synapses.

Each population consists of a cascade of linear and nonlinear modules, receiving average postsynaptic membrane potential *v*_*i*_ from other neural populations, the average synaptic connection constant represents the coupling between neural populations. Then, the membrane potential is converted into the average peak density of the neurons, and the sigmoid function is used to simulate the existence of inhibition and saturation, denoted by *z*_*i*_. Thus, *z*_*i*_ = *S*(*v*_*i*_). Changing the value could simulate the impulse responses of different synapses; the process is represented by *h*_*i*_(*t*).

By combining these two models, the brain’s processing of movement intention generation could be simulated and corresponding EEG signals could be generated. The complete lower limb movement intention generation model corresponds to the following set of differential equations:

Pyramidal neurons


(3) $$\left\{ \begin{array}{ccc} \frac{d\,y_{1}\,\left(t\right)}{d\,t} & = & x_{1}\,\left(t\right)\\ \frac{d\,x_{1}\,\left(t\right)}{d\,t} & = & G_{2}\,\omega_{2}\,z_{1}\,\left(t\right)-2\,\omega_{2}\,x_{1}\,\left(t\right)-\omega_{2}^{2}\,y_{1}\,\left(t\right)\\ z_{1}\,\left(t\right) & = & \frac{2\,e_{0}}{1+e^{-r\,v_{1}}}-e_{0}\\ v_{1}\,\left(t\right) & = & C_{12}\,y_{2}-C_{13}\,y_{3}\,\left(t\right)-C_{14}\,y_{4}\,\left(t\right) \end{array}\right.$$

Excitatory interneurons


(4) $$\left\{ \begin{array}{ccc} \frac{d\,y_{2}\,\left(t\right)}{d\,t} & = & x_{2}\,\left(t\right)\\ \frac{d\,x_{2}\,\left(t\right)}{d\,t} & = & G_{2}\,\omega_{2}\,\left(z_{2}\,\left(t\right)+\frac{u_{1}\,\left(t\right)}{C_{12}}\right)-2\,\omega_{2}\,x_{2}\,\left(t\right)-\omega_{2}^{2}\,y_{2}\,\left(t\right)\\ z_{2}\,\left(t\right) & = & \frac{2\,e_{0}}{1+e^{-r\,v_{2}}}-e_{0}\\ v\,2\,\left(t\right) & = & C_{21}\,y_{1}\,\left(t\right) \end{array}\right.$$

Slow inhibitory interneurons


(5) $$\left\{ \begin{array}{ccc} \frac{d\,y_{3}\,\left(t\right)}{d\,t} & = & x_{3}\,\left(t\right)\\ \frac{d\,x_{3}\,\left(t\right)}{d\,t} & = & G_{3}\,\omega_{3}\,z_{3}\,\left(t\right)-2\,\omega_{3}\,x_{3}\,\left(t\right)-\omega_{3}^{2}\,y_{3}\,\left(t\right)\\ z_{3}\,\left(t\right) & = & \frac{2\,e_{0}}{1+e^{-r\,v_{3}}}-e_{0}\\ v_{3}\,\left(t\right) & = & C_{31}\,y_{1}\,\left(t\right) \end{array}\right.$$

Fast inhibitory interneurons


(6) $$\left\{ \begin{array}{ccc} \frac{d\,y_{4}\,\left(t\right)}{d\,t} & = & x_{4}\,\left(t\right)\\ \frac{d\,x_{4}\,\left(t\right)}{d\,t} & = & G_{4}\,\omega_{4}\,z_{4}\,\left(t\right)-2\,\omega_{4}\,x_{4}\,\left(t\right)-\omega_{4}^{2}\,y_{4}\,\left(t\right)\\ \frac{d\,y_{4^{\prime }}\,\left(t\right)}{d\,t} & = & x_{4^{\prime }}\,\left(t\right)\\ \frac{d\,x_{4^{\prime }}\,\left(t\right)}{d\,t} & = & G_{2}\,\omega_{2}\,u_{4}\,\left(t\right)-2\,\omega_{2}\,x_{4^{\prime }}\,\left(t\right)-\omega_{2}^{2}\,y_{4^{\prime }}\,\left(t\right)\\ z_{4}\,\left(t\right) & = & \frac{2\,e_{0}}{1+e^{-r\,v_{4}}}-e_{0}\\ v_{4}\,\left(t\right) & = & C_{41}\,y_{1}\,\left(t\right)-C_{43}\,y_{3}\,\left(t\right)-C_{44}\,y_{4}\,\left(t\right)+y_{4^{\prime }}\,\left(t\right) \end{array}\right.$$

In the model, *y*_*i*_(*t*) represents the output of the corresponding neuron, and the overall output of the model is *v*_1_(*t*). Moreover, the subscript 4’ represents the negative self-loop of fast inhibitory interneurons. *C*_*ij*_ represents the synaptic constant from neuron *j* to neuron *i*. *G*_*i*_ represents the strength of the individual synapses and ω_*i*_ represents the reciprocal of the time constant. The sigmoidal function is centered on 0 and the parameters of the sigmoid function are represented by *e*_0_ and *r*.

## 3 Experiment

### 3.1 Experimental system overview

**FIGURE 4:**
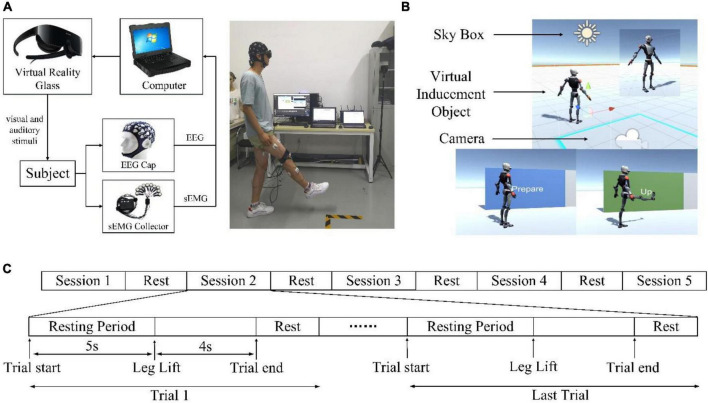
Experimental system. **(A)** The experimental platform. **(B)** Virtual scenarios. **(C)** Experimental Sequence Diagram.

In this study, an experimental platform was built to verify the above analysis. The experimental platform is built as shown in Figure 4A, including a VR module, an EEG signal acquisition module, an EMG signal acquisition module, and a host computer. HUAWEI VR Glasses were used to provide a VR environment. It adopts a dual fast LCD screen, with a field of view angle of 90 degrees and a binocular resolution of 3K and supports VR sound effects that move with the head. The advantage of VR glasses over VR helmets is that glasses do not need to be worn through the top of the head, which can reduce the adverse effects on EEG signals caused by friction between the device and the head. A Neuracle 32-channel EEG cap (Neusen W-EEG) was used to collect the EEG signals, and a Neuracle 16-channel EMG (NeuSen WM) acquisition instrument was used to collect the surface electromyography (sEMG) signals. The computer is used to receive and store the EEG and sEMG signals. Signal processing is carried out through MATLAB.

The construction of the virtual scenarios is shown in Figure 4B, which is implemented based on the Unity3D editor. The virtual character model induces the subjects to perform specified actions. The light source of the scene is the color of the sky, and the scene camera is bound to the virtual reality glasses. When the virtual characters in the scenarios start to move, the subjects need to follow them to move.

### 3.2 Subjects

A total of 12 college students were selected as subjects (marked as S1-S12), 10 male and 2 female, without any history of sensorimotor deficits or any psychological disorders. The demographic and physiological information of the subjects is summarized in Table 1. This study was approved by the Ethics Committee of Xi’an Jiaotong University (Ethics number: 2021-360). Before the start of the experiment, each of the subjects was introduced to the relevant tasks and signed the informed consent form.

**TABLE 1:** Demographic characteristics of the subjects.

Subjects	S1	S2	S3	S4	S5	S6	S7	S8	S9	S10	S11	S12	MEAN ± SDT
Age (years)	23	22	24	26	24	23	25	28	24	24	24	23	24.2 ± 1.5
Height (cm)	177	172	178	168	178	163	175	171	168	174	178	176	173 ± 4.7
Weight (kg)	71	73	74	63	64	53	67	68	57	72	64	69	66.3 ± 6.2

### 3.3 Experimental protocol

The experiment was completed in the Bio-Mechatronics and Service Robot Laboratory of Xi’an Jiaotong University. The leg lift movement is the subject of movement because it is one of the important basic movements in lower limb movement. This experiment is a control experiment. The experimental group used the VR induction system to induce the subjects to perform the action, the control group did not use it and changed to a single-tone prompt. To ensure the single variable principle of the controlled experiment, the VR induction system included the same single-tone prompt as the control group. All subjects were required to complete two experiments, one for the experimental group and the other for the control group. The experiments were conducted on the same day with a long break in between to minimize variability caused by factors such as fatigue. During the experiment, the subjects should always maintain a natural standing state. There were a total of 5 sessions in this experiment, and the sequence diagram is shown in Figure 4C. Each session consisted of 10 trials. In each trial, the subjects first kept still for 5 s and then performed actions according to the prompts. Signals around and before the start moment of the movement were monitored so that the movement execution was not limited to but did not exceed 4 s (2 s to raise the leg and 2 s to lower it). After the actions were completed, there was a short rest to prepare for the next trial. Each of the sessions was 135 s long with a break of 2–5 min between two consecutive sessions. Finally, each subject’s experimental and control group data were collected 50 times, respectively.

### 3.4 EEG data collection and movement evaluation

This experiment recorded the EEG signals of subjects during exercise. The measurement points of EEG electrodes under the international 10/20 system were FZ, FC1, FC2, CZ, C3, C4, CP1, CP2, and PZ. Before acquiring data, an appropriate conductive gel was applied to the scalp and ensure that the required impedance between the electrodes and the scalp was less than 5 kΩ. The sampling frequency was 1,000 Hz.

Lower limb movements were evaluated by surface EMG signals. Six surface EMG sensors were placed in the subjects’ rectus femoris, vastus lateralis, vastus medialis, semitendinosus, tibialis anterior, and gastrocnemius. These EMG data provided the changes in lower limb muscle activation during the experiment. The sampling frequency of the sEMG sensor was 1,000 Hz.

### 3.5 Data analysis method

The Cz channel of the EEG cap corresponds to the lower limb motor-related cortex, so we selected the EEG signal data of the Cz for analysis (Li et al., 2018; Romero-Laiseca et al., 2020). Two major neural phenomena can be captured with EEG in relation to movement intention when human lower limbs are moving, event-related desynchronization (ERD), and MRCP. ERD is recognized as a decrease in the α (μ) band power (8–13 Hz) and in the β band power (14–25 Hz) with movement (Qiu et al., 2016). MRCP is a low-frequency negative shift in the EEG recording that takes place approximately 0.5–2 s before the movement production. MRCP is readily masked by higher frequency activity, and its amplitude is usually between 5 and 30 μV (Shakeel et al., 2015). These two features could be calculated from the original EEG data. The collected EEG signals contain artifacts such as noise, EMG, and power frequency interference, which need to be filtered out before analysis. This study used empirical mode decomposition and independent component analysis for artifact removal.

Short-time Fourier transform (STFT) is used to analyze the collected data in time-frequency domain. It multiplies a time-limited window function before Fourier transforms the signal instead of Fourier transforming the entire signal. It assumes that the signal is stationary in the short time interval of the analysis window, and the spectrum of the signal at each moment in the time domain is obtained by moving the window function on the time axis. To observe the EEG responses of different states in the time domain, event-related spectral perturbation (ERSP) is used to analyze the power spectrum changes of EEG. The calculation of ERD features is as follows:


(7) $$E_{i,j}=\frac{1}{n}\,\sum_{k=1}^{n}s_{i,j,k}^{2}$$

(8) $$E_{b_{i}}=\frac{1}{m}\,\sum_{j=1}^{m}E_{i,j}$$

(9) $$E\,R\,D=\left(\frac{E_{i,j}-E_{b_{i}}}{E_{b_{i}}}\right)\times 100\%$$

Here, *s* represents the EEG signal. *i*, *j*, and *k* denote the trial number, epoch number, and sample number. *E* represents the mean power of the EEG data and *n* is the length of sub-epochs. *E*_*b*_ represents the baseline consisting of *m* epochs of each trial.

In STFT calculation, the choice of window function is crucial. The rectangular window has severe spectrum leakage. The BlackMan-harris window has an excessively wide main lobe that reduces the frequency resolution. The Hanning window has both good frequency resolution and less spectral leakage. Therefore, the Hanning window is used in this study. The EEG data were subdivided into 1-s-long epochs with a 200-ms overlap. Then, each epoch was processed for ERD extraction. Furthermore, the change of EEG power with frequency can be calculated by taking the ERSP power at different frequencies and then according to the mean value of the time dimension. Time-domain MRCP features could be extracted by filtering directly.

Moreover, a convolutional neural network (CNN) was used to detect the lower limb movement intention of the experimental group and the control group. Based on the convolutional neural network framework, this paper designed 13 convolutional layers, 5 pooling layers, 3 fully connected layers, and 1 normalization layer. The input data were filled with the “same” operation to ensure that the input and output sizes were the same after the convolution operation. The size of the convolution kernel was 3 × 3 and activated by the tanh function. The maximum pooling method was adopted for the pooling layer and the stride was 2 × 2.

## 4 Result

### 4.1 Simulation analysis

The central nervous system can always have rhythmic and spontaneous discharges without any external stimulation, so the input of the model can be simulated by a uniformly distributed random signal. The *u*_4_(*t*) during advanced brain processing is simulated by white Gaussian noise with mean 0 and variance 5. Referring to physiological knowledge, Sigmoid saturation (s^–1^) *e_0_* = 2.5, Sigmoid steepness (mV^–1^) *r* = 0.56, EEG signals of different states can be simulated by adjusting the connectivity constant and synaptic impulse response (Ursino et al., 2010).

**FIGURE 5:**
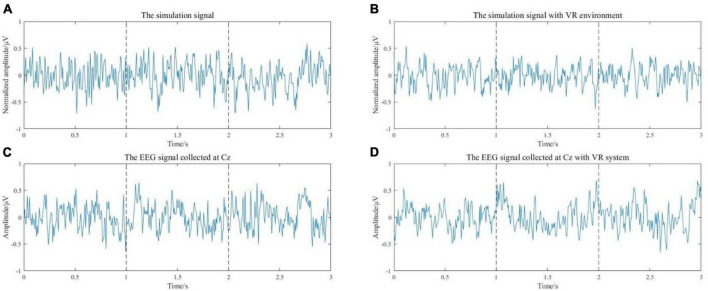
**(A)** The simulation results of the EEG signal during voluntary movement without external stimulus. **(B)** The simulation results of EEG signals during exercise with a VR environment. **(C)** The EEG signal of the Cz channel from S1. **(D)** The EEG signal of the Cz channel from subject 1 with VR induction system.

The simulation results of the EEG signal during voluntary movement without external stimulus are shown in Figure 5A. When there is an external stimulus from the VR system, the stimulus can be represented by a sine curve at the moment of movement intention generation. The model input for this case is a uniformly distributed random signal superimposed on a sinusoidal signal. The simulation results of EEG signals during VR-based motion are shown in Figure 5B. Figures 5C, D are the simulated signals corresponding to the actual acquired EEG signals of S1 at the Cz channel. The simulated signal in the time domain is similar to the actual collected signal. Then, the features related to movement intention need to be extracted and analyzed from the simulated EEG signals.

**FIGURE 6:**
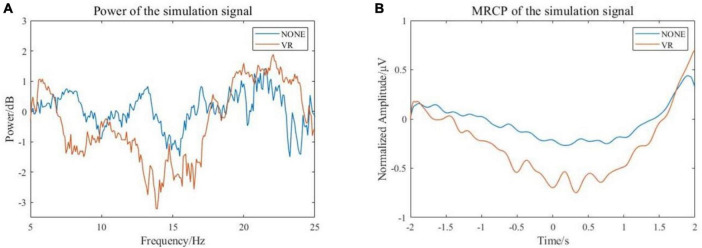
Characteristics of simulated EEG signal. **(A)** Frequency-domain features **(B)** Time domain features.

Event-related desynchronization and MRCP features of the simulated EEG signals were extracted. The calculated EEG signal power in α and β frequency bands is shown in Figure 6A. It can be seen that the power is decreased in both α and β bands, and the power decrease is more pronounced with the stimulus from the VR induction system. The results of filtering the low frequency (0–10 Hz) EEG signal are shown in Figure 6B. It can be seen that during the preparation and execution of lower limb movements, the amplitude of the EEG signal decreases first and then increases. The signal is more negatively shifted with the stimulus from the VR induction system. In general, comparing the simulated EEG signals of VR induction or not, both MRCP and ERD features are more obvious when there is movement with the external stimuli from the VR induction system. This means that the use of a VR induction system would be more conducive to detecting/predicting movement intention from EEG signals.

### 4.2 Neurophysiological data analysis

#### 4.2.1 ERD time-frequency analysis

**FIGURE 7:**
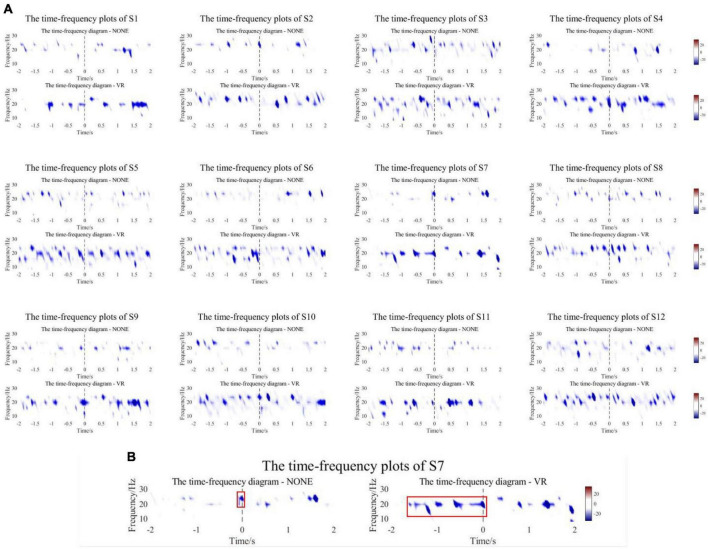
**(A)** The time-frequency plots of the experimental and control groups of subjects. **(B)** A representative subject. The starting position of the red box is the ERD onset time.

The collected EEG data of the experimental group and the control group were analyzed offline. Nerve conduction velocities ranged from approximately 50 to 70 m/s, and the nerve pathways involved in the reception of stimuli to intention generation were all on the millimeter scale. Therefore, the differences between the onset time of movement defined by the experimental group and the control group could be ignored. An epoch is 5 s before to 2 s after the motion. After preprocessing and filtering artifacts, 8–30 Hz fourth-order Butterworth band pass filtering was performed to obtain EEG signals in α and β frequency bands. The EEG data from −5 s to −2 s were regarded as the resting state, and the time-frequency plots of the 12 subjects at −2 s to 2 s were calculated and plotted based on this baseline. The signal was collected at the Cz channel. Figure 7 shows the time-frequency plots of the experimental and control groups of subjects.

Figure 7A shows that all subjects in the experimental group and control group had ERD phenomenon near the start of motion (−2∼2 s). The experimental group experienced a wider range of ERD phenomena and a more significant decrease in power compared to the control group. For quantitative analysis, the first occurrence −20 dB point is chosen as the point of initiation of ERD, and the ERD peaks of the experimental group and the control group were counted. As shown in Table 2, the mean onset time of ERD of subjects in the experimental group and control group was −1.723 ± 0.319 s and −1.336 ± 0.526 s, and the mean peak values were −30.874 ± 3.796 dB and −25.340 ± 2.511 dB. There was a significant difference in ERD Onset Time (*p* = 0.0191) and a highly significant difference in Peak Value (*p* = 0.0001) between the experimental and control groups. Most of the results yielded the same conclusion as the mean. All subjects in the experimental group had lower peaks than the control group, and most of the experimental group had ERD Onset Time earlier than the control group. However, the ERD onset time of the experimental group for S3 and S10 appeared slightly later than that of the control group, 0.051 s and 0.067 s, respectively, not exceeding 0.1 s. Nevertheless, the time-frequency plots clearly show that S3 and S10 produced a wider range of ERD phenomena in the experimental group, producing peaks that were 1.126 dB and 5.694 dB lower than in the control group, respectively. Figure 7B is a representative subject. The control group only showed a significant ERD phenomenon near the movement onset time, whereas the experimental group showed a wider range, especially before the start of the movement. The starting position of the red box is the ERD onset time.

**TABLE 2:** ERD onset time and peak value statistics.

Subject	ERD Onset Time (s)		Peak Value (dB)	
	**Experimental Group**	**Control Group**	**Experimental Group**	**Control Group**
S1	−1.133	−1.133	−30.237	−26.430
S2	−1.000	−0.933	−40.359	−31.113
S3	−1.809	−1.860	−27.831	−26.705
S4	−1.993	−1.867	−27.018	−23.066
S5	−2.015	−1.860	−31.345	−29.446
S6	−1.963	−1.603	−25.126	−24.293
S7	−1.600	−0.067	−30.964	−24.133
S8	−1.912	−1.037	−34.243	−24.264
S9	−1.860	−1.654	−32.932	−24.370
S10	−1.800	−1.867	−28.973	−23.279
S11	−1.654	−1.088	−29.010	−24.362
S12	−1.933	−1.067	−32.449	−22.621
MEAN ± STD	−1.723 ± 0.319	−1.336 ± 0.526	−30.874 ± 3.796	−25.340 ± 2.511

**FIGURE 8:**
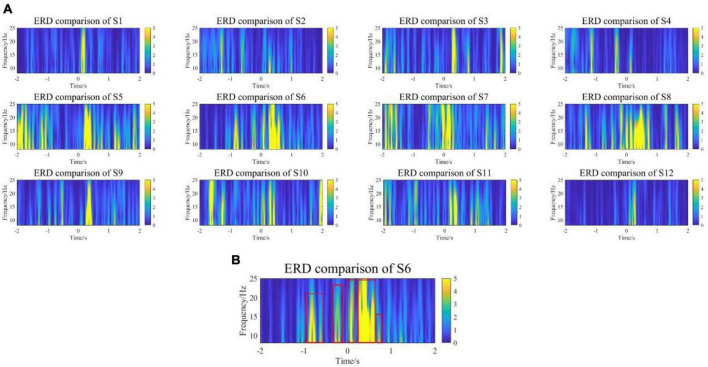
**(A)** The comparison results of ERD in the range of [–2,2] seconds between the experimental group and the control group. **(B)** A representative subject. The experimental group showed early significant differences in ERD even before the movement onset.

Additionally, comparing the statistical significance of the difference in ERD between the experimental group and the control group, the compared test was calculated at the 95% significance level, and the results are shown in Figure 8A. All subjects showed differences near the onset of exercise at both the α and β frequency bands, especially for the α frequency band. Surprisingly, except for S1, S3, and S12, the other 9 subjects showed differences in early stages. Figure 8B is a representative subject. The major significant blocks are marked in red rectangles. It showed early significant differences even before the movement onset. Early features could be conducive to pre-movement intention pattern detection.

**FIGURE 9:**
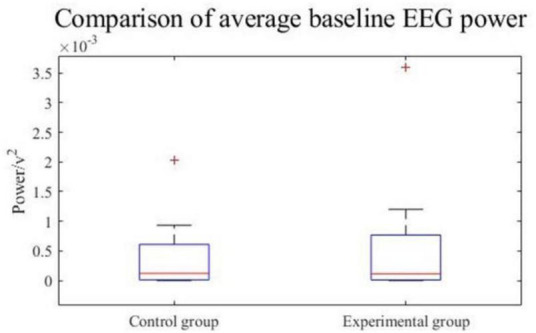
EEG baseline power.

Moreover, to investigate how baseline EEG was affected by experimental and control groups, baseline EEG powers from all channels at α and β frequency band (8–25 Hz) were calculated and averaged across all subjects. The data conformed to a normal distribution according to the Shapiro–Wilk test. The experimental group had a kurtosis of −0.9 and a skewness of 0.8, while the control group had a kurtosis of 4.9 and a skewness of 2.1. Figure 9 shows the boxplot of the EEG baseline power, the average EEG baseline power of the experimental group was 5.66 × 10^–4^ *v*^2^, and the control group was 3.89 × 10^–4^ *v*^2^. Although the average EEG baseline power of the experimental group was slightly higher than that of the control group, the results of the *t*-test showed that the experimental group and the control group were not significantly different.

Therefore, in the overall ERD time-frequency analysis, the baseline EEG power of the experimental group and the control group were consistent, which means that the VR induction system could not change EEG in the resting state. It could induce more obvious early ERD features to enhance the detectability of movement intention.

#### 4.2.2 Frequency domain power analysis

**FIGURE 10:**
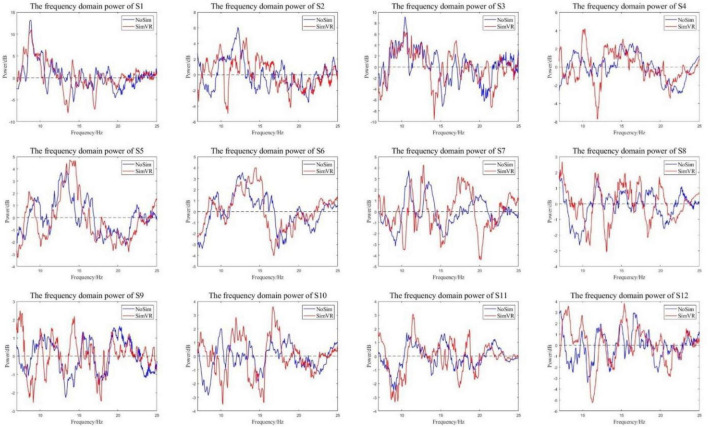
Energy curves in the frequency domain. The experimental group has more energy attenuation than the control group in α and β bands.

The ERSP power of all subjects was calculated, and the frequency domain power of the experimental group and the control group was analyzed. The graph of the change of EEG power with frequency is shown in Figure 10. The red line is the frequency domain power of the experimental group and the blue line is the frequency domain power of the control group.

All subjects in the control group and the experimental group had the same trend of frequency domain power change. The power was dropped in the α and β Frequency bands. From the perspective of power attenuation, throughout the entire frequency range, the experimental group of most subjects had greater attenuation than the control group. Because of the opposite results presented at certain moments in some smaller ranges (such as the power of S9 at around 13 Hz), the characteristic frequencies of each frequency band need to be analyzed. The frequency corresponding to the minimum value in the figure was the characteristic frequency; detailed data is shown in Table 3.

**TABLE 3:** Characteristic frequency in α and β band.

Subject	Experimental Group		Control Group	
	**α Band (Hz)**	**β Band (Hz)**	**α Band (Hz)**	**β Band (Hz)**
S1	13.0	17.0	14.0	20.0
S2	11.0	19.0	8.0	21.0
S3	14.0	19.0	14.0	19.0
S4	12.0	21.0	12.0	21.0
S5	10.0	21.0	11.0	20.0
S6	11.0	17.0	11.0	17.0
S7	10.0	20.0	9.0	22.0
S8	11.0	18.0	10.0	20.0
S9	9.0	18.0	13.0	18.0
S10	10.0	16.0	8.0	15.0
S11	9.0	17.0	9.0	17.0
S12	11.0	21.0	11.0	20.0
MEAN ± STD	10.9 ± 1.4	18.7 ± 1.7	10.8 ± 2.0	19.2 ± 1.9

**FIGURE 11:**
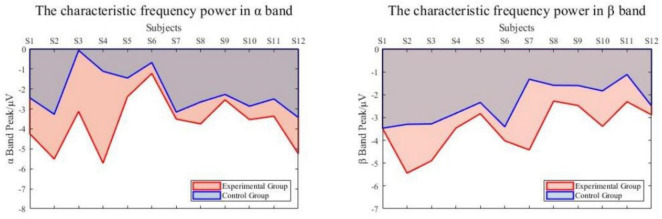
The power of characteristic frequency.

The characteristic frequencies of the α band experimental group and the control group were 10.9 ± 1.4 Hz and 10.8 ± 2.0 Hz, respectively. The characteristic frequencies of the β band were 18.7 ± 1.7 Hz and 19.2 ± 1.9 Hz. The characteristic frequencies of the experimental group and the control group were correlated (*R* = 0.94), so the experimental group did not change the main frequency of feature generation. The power of characteristic frequency is shown in Figure 11. The power of the experimental group decreased more than that of the control group. In the α band, the average peak power of the experimental group was −3.679 ± 1.281 dB, and that of the control group was −2.156 ± 1.039 dB. In the β band, the average peak power was −3.490 ± 0.984 dB and −2.379 ± 0.835 dB, respectively.

The frequency domain power analysis shows that the characteristic frequencies generated by each frequency band in the experimental group and the control group are correlated. That means the VR induction system could generate more significant power attenuation in EEG to enhance the detectability of movement intention.

#### 4.2.3 Time-domain MRCP feature analysis

**FIGURE 12:**
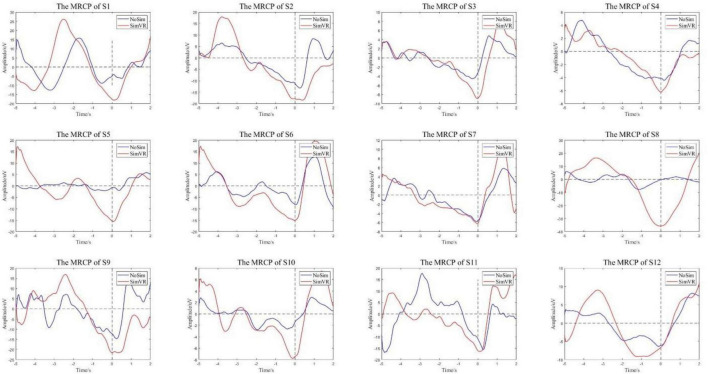
MRCP features. The experimental group showed obvious characteristics.

The collected EEG data of two working conditions were analyzed. An epoch is 5 s before to 2 s after the motion. The epoch was preprocessed to eliminate the artifacts, and 0.1–10 Hz filtering was performed to obtain low-frequency signals. Figure 12 compares the MRCP features extracted from subjects in the experimental group and the control group. The red line is the MRCP of the experimental group and the blue line is the MRCP of the control group. It could be seen that all subjects in the experimental group showed obvious MRCP characteristics, and the amplitude of this potential began to decrease 2–3 s before the onset of motion and then rebounded. In total, 11 subjects in the control group showed MRCP characteristics, only S5 did not show obvious MRCP characteristics.

**FIGURE 13:**
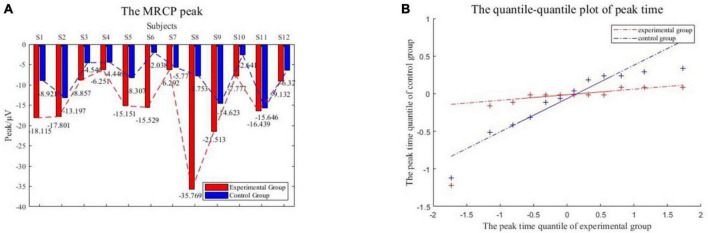
**(A)** The MRCP peak. **(B)** The quantile-quantile plot of peak time.

To compare the differences in the characteristics of the experimental group and the control group, the peak points of the MRCP characteristics were counted as shown in Figure 13A. The peak value of all experimental groups decreased more than that of the control group, with a significant statistical difference (*p* = 0.0082). The average peak value of the experimental group was −14.052 ± 8.757 μV and the control group was −7.855 ± 4.345 μV. Furthermore, comparing the peak time of the two groups, the time of peak appearance of the experimental group was −0.111 ± 0.343 s and the control group was −0.103 ± 0.412 s. Whether the peak time of the two groups came from the same distribution was verified through a quantile-quantile plot, as shown in Figure 13B, the red line is the distribution of the experimental group and the blue line is the distribution of the control group. The peak time of the experimental group and the control group follow different distributions, with statistical differences.

In time-domain MRCP feature analysis, the result means that the VR induction system could induce more significant MRCP features compared to enhancing the detectability of motion intention.

### 4.3 Offline classification analysis

To maintain the optimal performance of CNN classifiers, it is necessary to adjust the parameters. The principle is to enable the validation set to be classified with no more than 95% specificity while maintaining at least a sensitivity of 75%. The calculation formulas for sensitivity and specificity are as follows:


(10) $$\text{Sensitivity}=\frac{\mathrm{True}\,\mathrm{Positive}}{\mathrm{True}\,\mathrm{Positive}+\mathrm{False}\,\mathrm{Negative}}$$

(11) $$\mathrm{Specificity}=\frac{\mathrm{True}\,\mathrm{Negative}}{\mathrm{True}\,\mathrm{Negative}+\mathrm{False}\,\mathrm{Positive}}$$

Table 4 shows the offline classification results of 12 subjects in the experimental and control groups. Results for each subject were the mean after 10-fold cross-validation. The CNN classifier maintains a reasonable true positive detection rate while ensuring a minimal false positive detection. The accuracy rates for the experimental and control groups were 85 ± 2.85% and 82.22 ± 1.96%, respectively. Similarly, the sensitivity and specificity were 84.18 ± 2.84%, 82.1 ± 2.25% and 88.08 ± 4.21%, 84.8 ± 3.32%, respectively. The results showed that the accuracy (*p* = 0.0008) and specificity (*p* = 0.0097) of the experimental group were significantly higher than those of the control group, and the sensitivity (*p* = 0.0490) of the experimental group was significantly higher than that of the control group specificity. This means that the VR induction system could enhance the detectability of intentions.

**TABLE 4:** Classification results.

Subject	Accuracy (%)		Sensitivity (%)		Specificity (%)	
	**Experimental Group**	**Control Group**	**Experimental Group**	**Control Group**	**Experimental Group**	**Control Group**
S1	83.50 ± 1.29	82.41 ± 2.81	82.14 ± 2.42	83.47 ± 2.96	87.50 ± 2.55	86.23 ± 5.89
S2	85.20 ± 2.84	79.81 ± 0.60	80.91 ± 1.64	76.92 ± 0.70	91.86 ± 5.18	84.62 ± 1.37
S3	83.26 ± 2.06	80.49 ± 1.52	81.45 ± 1.85	82.61 ± 2.49	90.72 ± 4.87	77.78 ± 1.24
S4	81.33 ± 2.14	80.17 ± 1.09	79.37 ± 1.42	81.20 ± 1.29	83.84 ± 3.07	78.85 ± 2.31
S5	84.88 ± 3.65	82.87 ± 2.56	85.22 ± 3.15	86.78 ± 2.64	85.56 ± 5.24	84.21 ± 5.71
S6	83.41 ± 1.14	81.25 ± 1.11	84.35 ± 2.49	79.49 ± 1.43	86.67 ± 2.07	85.71 ± 1.76
S7	84.50 ± 2.45	81.63 ± 1.67	83.04 ± 2.54	80.91 ± 1.76	86.36 ± 3.55	84.88 ± 3.14
S8	86.73 ± 3.35	84.91 ± 3.59	85.45 ± 3.18	83.19 ± 3.79	90.70 ± 4.21	88.17 ± 4.84
S9	86.34 ± 3.05	83.00 ± 2.88	84.35 ± 2.42	82.14 ± 2.55	88.89 ± 4.46	86.36 ± 4.47
S10	90.50 ± 6.74	84.18 ± 1.82	90.32 ± 6.13	81.82 ± 2.07	90.72 ± 8.06	88.37 ± 2.40
S11	85.85 ± 2.94	83.96 ± 1.68	86.96 ± 3.70	85.71 ± 2.91	85.56 ± 2.59	86.02 ± 3.78
S12	84.50 ± 2.52	81.94 ± 2.19	86.61 ± 3.20	80.99 ± 2.36	88.64 ± 4.65	86.32 ± 4.48
MEAN ± STD	85.00 ± 2.85	82.22 ± 1.96	84.18 ± 2.84	82.10 ± 2.25	88.08 ± 4.21	84.80 ± 3.32

### 4.4 Comparison of EMG activity

**FIGURE 14:**
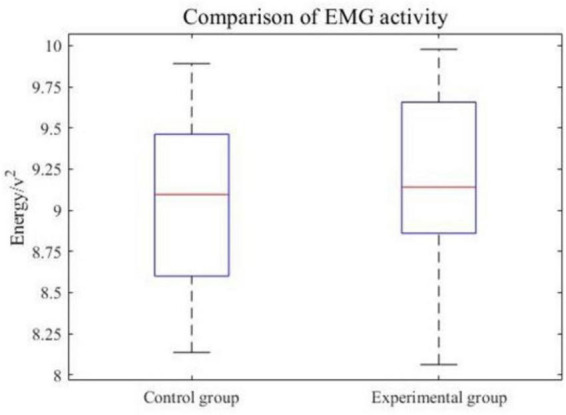
Comparison of EMG activity.

To further analyze the potential impact of the VR environment adopted by the experimental group on motor activity, muscle activation in the experimental and control groups was compared. Muscle activation can reflect the overall level of the movement execution process. The rectus femoris is an important muscle that reflects lower limb movements, therefore the EMG signal at the rectus femoris is used to calculate muscle activation. The EMG power average for each trial of the subjects was calculated to obtain a global statistical representation of muscle activation energy. Figure 14 shows a boxplot of the EMG activity from all subjects. It can be seen that there was no significant difference in the EMG activity of the subjects in the experimental group and the control group. This indicates that the movement execution of the experimental group and the control group in the experiment was consistent.

## 5 Discussion

The above results show that the motion of the subject with the VR induction could enhance the detectability of movement intention via EEG signals compared with the general situation.

Most existing BCI control technologies have some shortcomings because they are based on empirical evidence or experimental results. Thus, mathematic modeling can further our understanding of the physiological mechanisms for the responses of EEG behavior. By simulating the lower limb movement intention generation model in different conditions, the results show that the EEG signals related to movement intention had the corresponding features in the time and frequency domain, whatever the conditions. However, the VR induction could increase the significance of features. This provides a basis for feature selection during subsequent classification. The proposed model analyzes the mechanism of the brain’s movement intention in sections and discusses the coupling of multiple neuronal clusters, which is of great practical significance for the study of the brain functional network. Movement intention with the VR induction enhanced mechanism provides a new method for the active regulation of nerves in patients’ clinical rehabilitation.

In our experimental work, with ERD time-frequency analysis, we found that subjects’ lower limb movement with VR induction leads to a more prevalent ERD phenomenon, which has a better time-frequency resolution. Significant peak drops could make it easier to detect. Some results showed a certain early saliency compared with the general situation, which indicates that it is easier to be detected before the movement occurs. However, the baseline power is not affected by different scenes. This suggests that VR induction could be an effective way to detect motion intention using EEG signals.

Moreover, the ERD in the β band is more significant than that in the α band, whether with VR induction or not. Further frequency domain power analysis demonstrates that the power of α and β bands had both decreased, and the characteristic frequencies were similar. Compared to the characteristic frequency power in the α and β bands, the power drop in the β band is obviously greater than in the α band. When using the VR induction system, the decline was enhanced in both the α and β bands, significantly so in the α band. This means that peak α power and peak β power could be used as a combined feature in movement intention detection with VR induction and peak β power was highly sensitive to detection.

Time domain MRCP feature analysis shows that the features generated are more obvious with VR-based motion, and the amplitude drops more. The MRCP consists of the readiness potential (RP), motor potential, and movement-monitoring potential (MMP). RP is considered to reflect the planning or preparation of the movement and motor potential and MMP is thought to reflect movement execution and control of performance. RP feature enhancement could help to decode pre-movement EEG signals.

The enhancement of the above features is of great significance for clinical practical applications. The offline classification results prove that the VR induction system could improve the detectability of the BCI system. The analysis is based on Cz sampling points and the region around Cz corresponds to lower limbs (Blanco-Diaz et al., 2023). In addition, there is no significant difference between the experimental group and the control group in muscle activation energy during movement. This means the VR induction system could improve the movement-related features of EEG signals and further enhance the detectability of lower limb movement intention based on BCI.

Furthermore, the enhancement of the VR system on the brain is multifaceted and may contribute to brain nerve remodeling (Namazi et al., 2021; Tan et al., 2021). The mathematical model we have developed is based on the physiological mechanisms of the brain information processing process. The focus of this study is on movement intention, so the simulation study was carried out for the EEG signals related to movement intention. During the simulation of the model, the intervention of the VR system was considered to have an effect on the selective attention process during brain information processing. When the input signal was changed, only the synaptic constants between the neuronal clusters were changed, and there was no specific change in the structure of the model, so we believe that the model has the ability to migrate to other similar tasks, which will be studied in the future. This study was conducted on EEG signals related to movement intention. Simulation results based on the model guided the analysis of the experimental data, and the consistent conclusions obtained from the experimental and simulation results can illustrate the validity of the model.

In summary, the time and frequency domain characteristics of subjects’ EEG signals induced by VR are more obvious, the features appear earlier, and the intention detection accuracy is higher. Therefore, the intervention of VR induction can significantly improve the detectability of movement intention.

## 6 Limitations

Virtual reality scenarios make user interaction more natural when using rehabilitation robots, while immersion in the VR environment requires users to wear VR glasses or VR headsets, which may cause discomfort or visual fatigue. However, current research suggests that the help of an immersive VR environment can at least help increase movement intention, which in turn promotes neurorehabilitation. It could help people with impaired athletic ability to regain lost athletic ability to a certain extent with less effort and in less time. Additionally, to reduce the potential for hazards when moving while wearing a VR device, appropriate safety protocols should be designed and implemented so that users can be alerted to any tasks that require attention. This raises another interesting research question that should be investigated in future studies.

The limitation of this study is that the improved neural mass model in our study from a macroscopic point of view described the EEG generation mechanism of movement. Although the features of the simulated EEG signals had the same result as the experiment result, it still lacks some physiological validation. This should be refined in subsequent studies. In addition, EEG-EMG coherence analysis can respond to a certain extent to signal changes, which is a worthy topic for future research and contributes to the study of movement intention recognition methods based on the fusion of EEG and EMG signals. Furthermore, the experiment did not use subjects with lower limb dyskinesia, which will be resolved in future studies, but this study has been able to prove the effectiveness of VR induction in movement intention enhancement. The generation of active movement intention is the premise of active rehabilitation, and it is of great significance to improve the detectability of EEG related to the active motion intention of patients. To further improve the results, extensive research should be carried out on the details of the VR paradigm in the future.

## 7 Conclusion

In order to solve the problems of weak movement intention and low recognition accuracy in the rehabilitation process of people with lower limb motor dysfunction, this paper studied whether VR induction could enhance the detectability of lower limb active movement intention. The active movement intention generation process of individuals with lower limb dysfunction was analyzed, and an EEG generation theoretical model was established. A comparative experiment was conducted on 12 healthy subjects. Through simulation research and experimental results analysis of EEG signals, the multiple features were enhanced when subjects used VR induction so that VR induction could work as a tool to enhance the distinguishability of lower limb active movement intentions from EEG signals. Furthermore, offline classification proves that VR induction could enhance the detectability of movement intention. However, further work is necessary to quantify the effect of VR scenario stimuli on neural signals. Moreover, advanced signal processing and learning techniques could be employed to further enhance the results. In general, the current results show promising insights into VR scenarios and their effect on movement intention, preparation, and execution.

## Data availability statement

The original contributions presented in the study are included in the article/supplementary material, further inquiries can be directed to the corresponding author.

## Ethics statement

The studies involving humans were approved by the Ethics Committee of Xi’an Jiaotong University. The studies were conducted in accordance with the local legislation and institutional requirements. The participants provided their written informed consent to participate in this study. Written informed consent was obtained from the individual(s) for the publication of any potentially identifiable images or data included in this article. All the experiments were conducted in accordance with the Declaration of Helsinki.

## Author contributions

RD: Writing—original draft, Writing—review and editing. XZ: Writing—review and editing. HL: Writing—original draft, Writing—review and editing. GM: Writing—review and editing. AZ: Writing—review and editing. XS: Writing—review and editing. CH: Writing—review and editing.
